# Telomerase-positive circulating tumor cells are associated with poor prognosis via a neutrophil-mediated inflammatory immune environment in glioma

**DOI:** 10.1186/s12916-021-02138-7

**Published:** 2021-11-12

**Authors:** Wen Zhang, Tiancheng Qin, Zhenrong Yang, Liyuan Yin, Changyun Zhao, Lin Feng, Song Lin, Binlei Liu, Shujun Cheng, Kaitai Zhang

**Affiliations:** 1 Department of Immunology, National Cancer Center/National Clinical Research Center for Cancer/Cancer Hospital, Chinese Academy of Medical Sciences and Peking Union Medical College, Beijing, 100021 China; 2 State Key Laboratory of Molecular Oncology, Department of Etiology and Carcinogenesis, National Cancer Center/National Clinical Research Center for Cancer/Cancer Hospital, Chinese Academy of Medical Sciences and Peking Union Medical College, Beijing, 100021 China; 3 Lung Cancer Center, West China Hospital, Sichuan University, Chengdu, 610041 China; 4 Chongqing Diatech Biotechnological Limited Company, Chongqing, 400020 China; 5 Department of Neurosurgery, Beijing Tiantan Hospital, Capital Medical University, Beijing, China; 6 National Clinical Research Center for Neurological Diseases, Center of Brain Tumor, Beijing Institute for Brain Disorders and Beijing Key Laboratory of Brain Tumor, Beijing, 100070 China; 7 National “111” Center for Cellular Regulation and Molecular Pharmaceutics, Key Laboratory of Fermentation Engineering (Ministry of Education), Hubei Provincial Cooperative Innovation Center of Industrial Fermentation, College of Bioengineering, Hubei University of Technology, Wuhan, 430068 China

**Keywords:** Circulating tumor cells, Telomerase, Prognosis, Glioma, Neutrophils, Immune macroenvironment, Neutrophil extracellular traps

## Abstract

**Background:**

Gliomas are the most common aggressive cancer in the central nervous system. Considering the difficulty in monitoring glioma response and progression, an approach is needed to evaluate the progression or survival of patients with glioma. We propose an application to facilitate clinical detection and treatment monitoring in glioma patients by using telomerase-positive circulating tumor cells (CTCs) and to further evaluate the relationship between the immune microenvironment and CTCs in glioma patients.

**Methods:**

From October 2014 to June 2017, 106 patients newly diagnosed with glioma were enrolled. We used the telomerase reverse transcriptase CTC detection method to detect and analyze the CTC statuses of glioma patients before and after surgery. FlowSight and FISH confirmed the CTCs detected by the telomerase-based method. To verify the correlation between CTCs and the immune response, peripheral white blood cell RNA sequencing was performed.

**Results:**

CTCs were common in the peripheral blood of glioma patients and were not correlated with the pathological classification or grade of patients. The results showed that the presence of postoperative CTCs but not preoperative CTCs in glioma patients was a poor prognostic factor. The level of postoperative CTCs, which predicts a poor prognosis after surgery, may be associated with neutrophils. RNA sequencing suggested that postoperative CTCs were positively correlated with innate immune responses, especially the activation of neutrophils and the generation of neutrophil extracellular traps, but negatively correlated with the cytotoxic response.

**Conclusions:**

Our results showed that telomerase-positive CTCs can predict a poor prognosis of patients with glioma. Our results also showed a correlation between CTCs and the immune macroenvironment, which provides a new perspective for the treatment of glioma.

**Supplementary Information:**

The online version contains supplementary material available at 10.1186/s12916-021-02138-7.

## Background

Glioma is the most common aggressive cancer in the central nervous system (CNS) and represent 75% of primary intracranial tumors in adults [1]. Many previous studies have shown that after surgery combined with radiotherapy and chemotherapy, glioma still has an aggressive tendency toward intracranial recurrence, and the growth rate after recurrence is accelerated, worsening patient prognosis [2, 3]. Data have shown that the median recurrence period of glioblastoma (GBM) is 8–12 months, the median recurrence period of stromal glioma is 18–36 months, and the median overall survival (OS) of recurrent glioma is only 25–35 weeks [4, 5]. Therefore, considering the poor prognosis of gliomas and the difficulty in monitoring glioma response and progression, it is necessary to establish an approach for evaluating the progression or survival of patients with glioma.

Advancement in the development of molecular biology techniques for glioma has led to the discovery of a series of biomarkers with practical value for diagnosis, differential diagnosis, and treatment [6, 7]. Although these molecular markers have made great breakthroughs in the diagnosis and treatment of glioma, patient prognosis is still not optimistic, and there is no molecular biomarker that can accurately distinguish the subtypes of glioma or predict prognosis. The rapid development of liquid biopsy technology, especially relevant studies on circulating tumor cells (CTCs), shows that this technology is helpful for the clinical treatment and prognosis prediction of GBM patients [8, 9]. Although CTC studies in glioma patients show potential clinical applications, there are still some questions that need to be answered, such as whether CTCs can represent the main nature of tumor cells to correctly reflect the clinical behavior of GBM patients with recurrence and metastasis and provide more accurate information for individual treatment and prognosis prediction.

Based on previous studies [10, 11], a viable CTC detection method based on human telomerase reverse transcriptase (TBCD) was used in this study to evaluate the preoperative and postoperative CTC status of glioma patients. Next, the correlation between CTCs and clinical indicators was studied, and recurrence and prognosis were assessed. To better illustrate the clinical application value of CTCs in glioma, we analyzed the significance of the correlation between CTCs and patients’ macroimmunity in depth and proposed that the postoperative macroimmunity status of patients is closely related to CTCs and patient prognosis. To the best of our knowledge, this study is the first on the correlation between macroimmunity and CTCs in glioma patients, and we believe that our results can provide a new perspective for the clinical application of CTCs in glioma and even the treatment of glioma.

## Methods

### Collection of patient and clinical information

In this study, patients and healthy control volunteers all provided written informed consent, and peripheral blood collections were approved by the Ethics Committee of the Cancer Hospital, Chinese Academy of Medical Sciences. From October 2014 to June 2017, 106 patients newly diagnosed with glioma were enrolled, and the clinical information of the included patients was provided by the Department of Neurosurgery, Beijing Tiantan Hospital. Furthermore, another 31 patients were enrolled according to the screening conditions for RNA sequencing research. The clinical characteristics of the enrolled patients are shown in Table 1. All methods were performed in accordance with the approved guidelines.

**Table 1:** Patient Characteristics

No. of patients	106
Age (yr)	
Mean (range)	42.63(13-70)
Sex (%)	
Male	37(34.9)
Female	69(65.1)
Pathology (%)	
A	19(17.9)
O	5(4.7)
AO	8(7.5)
OA	29(27.4)
AOA	15(14.2)
GBM	30(28.3)
WHO Grade (%)	
1	2(1.9)
2	37(34.9)
3	31(29.2)
4	36(34.0)
KPS (%)	
60	2(1.8)
70	10(9.5)
80	25(23.6)
90	66(62.3)
100	3(2.8)

*A* astrocytoma; *O* oligodendroglioma; *AO* anaplastic oligodendroglioma; *OA* oligoastrocytoma; *AOA* anaplastic oligoastrocytoma; *GBM* glioblastoma multiforme; *KPS* Karnofsky performance status.

### Blood samples

To explore the significance of tumor reduction surgery on CTCs of glioma, we detected the patients’ CTC counts before surgery and compared them with their CTC counts at 7 days post-operation. All patients with gliomas were first treated and underwent surgery, and long-term follow-up was conducted after the surgery. Four milliliters of blood was collected from eligible patients or healthy donors (aged 20–50 years) in K2E (EDTA) tubes and stored at 4 °C in the lab within 2 h. The peripheral blood of another 31 glioma patients was collected for WBC RNA sequencing, and postoperative CTCs were also detected.

### Cell and virus agent

The human glioma cell line U251 was obtained from the Cell Resource Center, Peking Union Medical College, and cultured using minimum essential medium with Earle's balanced salts (MEM-EBSS) containing 10% fetal bovine serum (FBS) at 37 °C in 5% CO_2_.

In this study, the oHSV1-hTERT-GFP virus was constructed as described in a previous study [10]. Based on the selectivity of telomere activity, the oHSV1-hTERT-GFP virus captured telomere activity that is widely expressed in cancer cells and expressed green fluorescent protein (GFP).

### Identification of samples with glioma

The peripheral blood of patients with glioma was collected (4 ml) and prepared with K_2_E-EDTA anticoagulant tubes within 2 h of isolation. Details of the TBCD that we used have been described previously [10, 11]. APC-anti-human CD45 (clone: HI30, Invitrogen, USA) was added to 10^7^ cells/sample and incubated at room temperature for 30 min. Flow cytometry (BD, USA) was used to detect the GFP+/CD45- cell population, which was recorded as a positive result (Additional file 1: Fig S1A).

### GFP fluorescence observation

U251 cells or patient samples were transfected with the viral agent and cultured in a 6-well plate for 24 h, and the cells were observed under a fluorescence microscope (Olympus).

### Fluorescent in situ hybridization (FISH)

FISH was performed using a Vysis LSI 1p36 Microdeletion Region Probe (Abbott). DNA-FISH was applied to investigate the presence of CTCs. Peripheral blood samples (4 ml) from glioma patients were treated with a standard CTC testing procedure with some modifications. Peripheral blood samples were transfected with oHSV1-hTERT-GFP and cultured for 24 h. The cells were selected with an MS column by using anti-CD45 microbeads. The unlabeled cells were collected on a slide, and the slides were observed under a fluorescence microscope, confirming that the GFP+ cells were on the slide. Probes were used according to the manufacturer’s protocol. The prepared slides were scanned by fluorescence in situ scanner.

### Identification of CTCs using ImageStreamX®

APC-CD56 antibody (clone: CMSSB, Invitrogen, USA) and Alexa Fluor 405 CD45 antibody (clone: HI30, Invitrogen, USA) were used to identify CTCs. The CTC images were detected by the ImageStreamX® Mark II system (Amnis) (Additional file 1: Fig S1B). CD45/GFP+/CD56+ cells in the blood samples were considered CTC-positive cells.

### Next-generation sequencing (RNA-seq) and data processing

Peripheral blood was obtained from healthy controls (*n*=23) and untreated glioma patients (*n*=31). After red blood cell lysis buffer (Qiagen) was used to remove red blood cells, the total RNA of the remaining WBCs was extracted using TRIzol (Invitrogen). RNA-seq libraries were prepared from qualified samples using the NEBNext® Ultra™ RNA Library Prep Kit for Illumina (NEB) according to the manufacturer's instructions.

Clean reads in FASTQ files were quantified against an Ensembl catalog (GRCh37) at the transcript level using Salmon and aggregated to the gene level using tximport; transcripts per million reads (TPM) values were obtained with Salmon gene expression quantification software, and unexpressed genes were removed.

We defined genes positively correlated with CTCs as having a correlation coefficient *R* > 0.4, *p* < 0.05 (Cytoscape software) [12], which presented biological role specificity for the most significant differentially expressed gene transcripts based on their functional and pathway enrichment probes. The GO database [13] was used. Immune cells with differentially expressed genes were distinguished by using the “DESeq2” R package; genes with fold change > 2 and *p* < 0.05 were defined as differentially expressed genes. Heatmaps were made by using the “Pheatmap” R package, and survival curves were evaluated with the log-rank test (two-tailed) by the Kaplan-Meier method. GO analysis and hierarchy relation analysis were conducted with the “topGO” R package.

For each given gene list, pathway and process enrichment analyses were carried out using Metascape [14] with the following ontology sources: KEGG Pathway and GO Biological Processes. *Z*-score analysis was conducted with the “GOplot” R package. All genes in the genome were used as the enrichment background. Terms with a *p* value < 0.01, a minimum count of 3, and an enrichment factor > 1.5 (the enrichment factor is the ratio between the observed counts and the counts expected by chance) were collected and grouped into clusters based on their membership similarities.

GSEA was performed using the “GSVA” package, which uses the C7 immunologic signature gene sets and Hallmark gene sets downloaded from the Molecular Signatures Database (MSigDB) [15, 16]. Estimating the Proportion of Immune and Cancer cells (EPIC) [17] was used to analyze peripheral blood cell distribution.

GSEA enrichment analysis between immune-related gene sets and NETS was conducted by enrichment score in peripheral blood genes compared with different CTCS levels in the two groups. The immune gene set was also derived from a publication published by Bindea G. et al. [18]. Using the gene expression signatures of NETs, the GSEA implementation method relies on the R package “GSEABase,” resulting in an enrichment score ch gene set and NE values for the immune-related phenotypes of the 20 peripheral blood transcriptomes generated using the above methods.

### Statistics

The data were analyzed by generalized linear regression ANOVA in a cell line model and clinical samples. The cutoff value was determined by the Youden index and ROC curve. The Spearman correlation coefficient was used to assess correlations with clinical information. The Kaplan-Meier method was used to estimate the event time distribution, and the log-rank test was used for comparison. A multivariate Cox proportional hazards regression model was used to check the clinical descriptive information. SPSS 24.0 and GraphPad Prism 7.0 were used for data analysis. All the statistical tests were two-sided, with p values of less than 0.05 considered significant.

## Results

### TBCD effectively detected glioma CTCs in glioma patients

Previous studies have shown that TBCD can effectively detect CTCs in patients with different types of tumors. Therefore, we first verified the effectiveness and accuracy of this method in detecting glioma patients’ CTCs in vitro. As shown in Additional file 1: Fig S2A, the U251 cells expressed GFP 12 h after oHSV1-hTERT-GFP transduction, and a strong positive signal for GFP was detected in almost all U251 cells at 24 h after transduction. Variable numbers of U251 cells were spiked into 4-ml blood samples from healthy donors and analyzed using TBCD to confirm the accuracy. The recovery rate of U251 cells was 67.5–85.7%. The number of CD45-/GFP+ cells and the number of glioma cells that spiked in showed a strong correlation (*r*^2^ = 0.9871, 95% CI, 0.7213 to 0.7767) (Additional file 1 Figure: S2B). This result suggested that this method can reflect the number of CTCs in the in vitro model well.

**Fig. 1:**
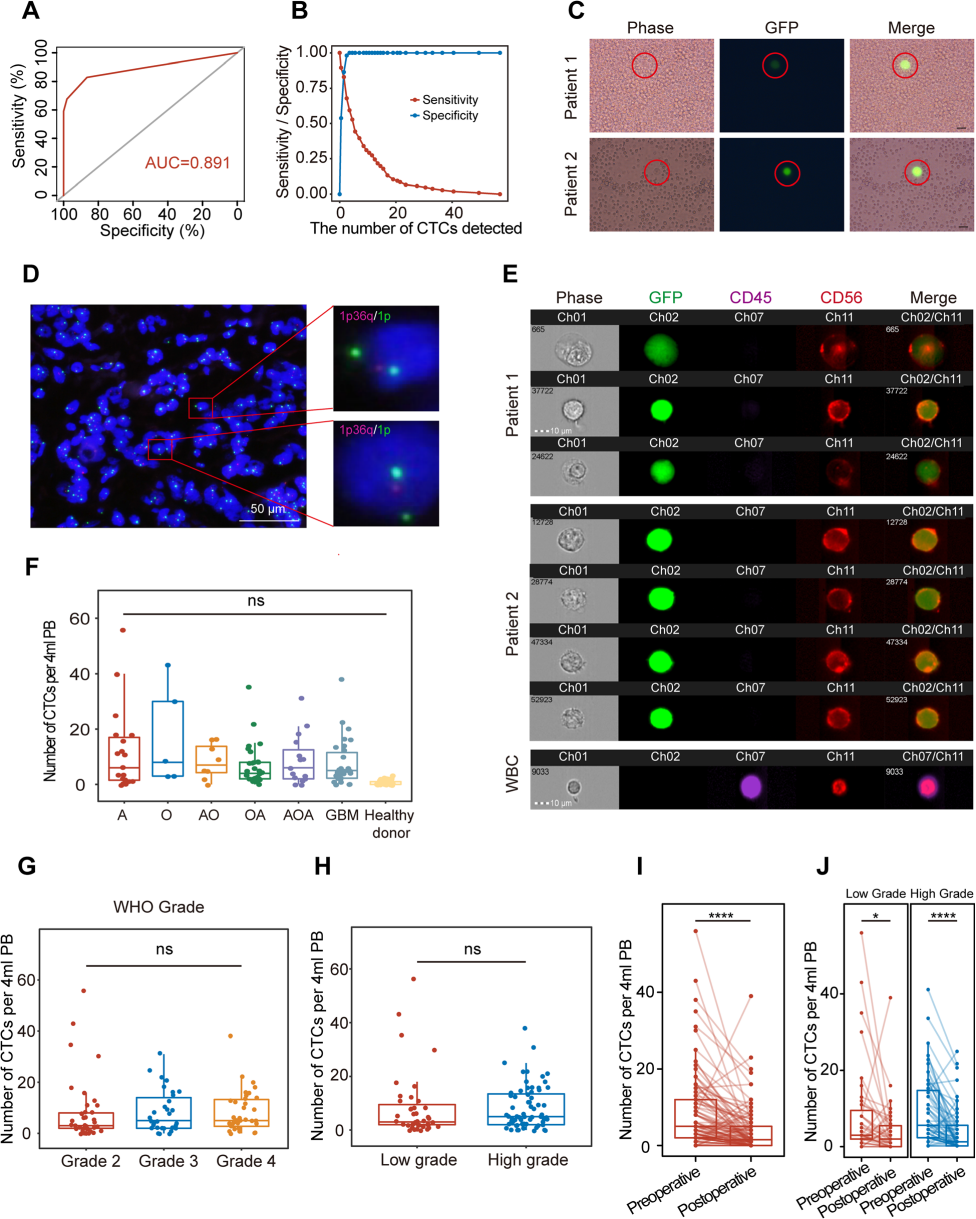
TBCD effectively detected glioma CTCs in glioma patients. **A** ROC analysis for the threshold detection of CTCs (AUC= 0.892). **B** Youden index analysis of TBCD according to previous findings in the group (*n*=158) with 52 healthy donors and 106 glioma patients. **C.** Typical CTCs in the peripheral blood leukocytes of the blood sample were visualized using GFP expression. Scale bar, 10 μm. **D** Images of CTCs in GBM patients revealed by DNA-FISH. Red: 1p; Green: 1q. Scale bar, 50 μm. **E** Typical CTC images of ImageStream® flow cytometry analysis. Ch01: bright field; Ch02: GFP (green); Ch07: CD45 (purple); Ch11: CD56 (red). CTCs were identified as CD45^−^/GFP^+^/CD56^+^ or CD45^−^/GFP^+^/CD56^−^, and WBCs were CD45^+^/CD56^+^. Scale bar, 10 μm. **F** Number of CTCs in 6 pathologic types of gliomas. A, astrocytoma; O, oligodendroglioma; AO, anaplastic oligodendroglioma; OA, oligoastrocytoma; AOA, anaplastic oligoastrocytoma; GBM, glioblastoma multiforme. **G** Comparison of CTC counts in grade II (*n*=36), grade III (*n*=26), and grade IV (*n*=26) gliomas. **H** Comparison of CTC counts between low-grade (WHO grades 1 and 2, *n*=38) and high-grade (WHO grades 3 and 4, *n*=52) gliomas. **I** The number of CTCs detected in preoperative and postoperative patients. ****, *p*< 0.0001. **J** The number of CTCs detected in the low-grade group and the high-grade group. *, *p*=0.044; ****, *p*< 0.0001

Then, we analyzed 106 whole blood samples from patients with different stages of glioma aged 13 to 79 years (mean = 42.63 years). The details of the patient characteristics for CTC detection are listed in Table 1. Based on the ROC analysis of CTC data from 52 healthy donors and 106 patients with glioma (Fig. 1A), the AUC of this model was 0.892 (95% CI, 0.843–0.941). The threshold was the cutoff point with the Youden index (sensitivity=0.83, specificity=0.865), defined as 1 CTC per 4 ml of blood (Fig. 1B). The average number of detected CD45−/GFP+ cells was 8.453±0.955 cells/4 ml for patients with glioma, while the average number of detected CD45−/GFP+ cells was 0.615±0.107 cells/4 ml for healthy donors in the control group (*p* < 0.0001, Additional file 1 Figure: S3).

Then, circulating glioma cells were imaged using green fluorescence (Fig. 1C). The results showed that CTCs existed in the peripheral blood and could be detected by TBCD. One of the most widely used markers for glioma is 1p/19q LOH, and we used FISH to visualize CTCs. The specimens we detected had the typical 1p deletion (Fig. 1D). To ensure that there were no false-positive cells from the WBCs in the sample, we used TBCD for clinical blood samples subjected to flow imaging. Antibodies against CD45 and CD56 were used to identify cancer cells [19]. As shown in Fig. 1E, GFP+/CD45−/CD56+ cells were identified as glioma cells, and CD45+/CD56+ cells were identified as NK cells (Additional file 1 Figure: S4). These results indicate that TBCD can efficiently and accurately detect CTCs in glioma patients.

### CTCs were not related to the clinical features of glioma patients

As shown in Table 1, CTCs were found in all 6 pathologic subtypes of gliomas, including astrocytoma (A), oligodendroglioma (O), anaplastic oligodendroglioma (AO), oligoastrocytoma (OA), anaplastic oligoastrocytoma (AOA), and GBM. Based on the results of ROC analysis, we defined patients with a test value of ≤ 1 cell/4 ml as a negative result. The total incidence of CTCs was 83.02% (88/106) before surgery. The variation in preoperative CTC counts ranged from 0 to 56 per 4 ml of peripheral blood (Additional file 2). However, no significant difference in CTC counts was observed between different pathologic subtypes of gliomas (Fig. 1F–H). The positive rate of preoperative CTCs ranged from 62.5% to 100% (Additional file 1 Figure: S5). However, there were no significant differences between different grades in preoperative CTC counts (Additional file 3). These results suggested that CTCs were widely present in the peripheral blood of glioma patients, and their count was not related to the pathological classification or grade of patients before surgery.

**Fig. 2:**
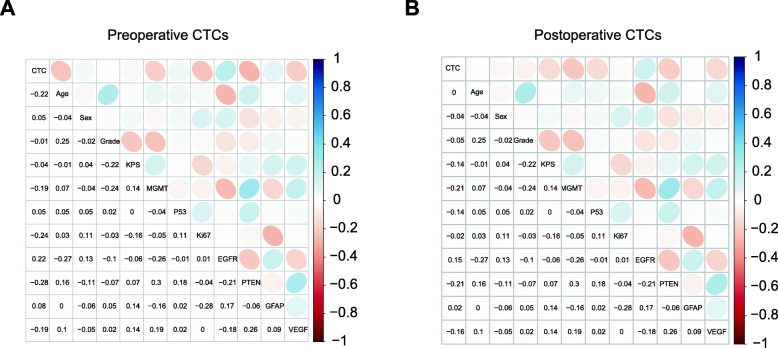
Postoperative CTCs as a prognostic biomarker. **A** Correlation heatmap (Pearson correlation) of CTCs and clinical parameters. Positive correlations are shown in cool colors, while negative correlations (anticorrelations) are shown in hot colors. **B** Correlation heatmap of postoperative CTCs and clinical parameters. Positive correlations are shown in cool colors, while negative correlations (anticorrelations) are shown in hot colors

Subsequently, we compared the changes in CTCs before and after surgery in glioma patients. Notably, the CTC counts of patients before and after surgery were significantly different (*p* < 0.0001) (Fig. 1I), but there was no correlation between preoperative CTCs and postoperative CTCs (Pearson *r* = 0.3642). As shown in Fig. 1J, the CTC counts of the high-grade group and low-grade group were significantly different (*p* < 0.0001 and *p* = 0.044, respectively) preoperatively and postoperatively. However, as shown in Fig. 2A and B, there were also no correlations between preoperative/postoperative CTCs and patient clinical features or tumor markers (MGMT, P53, Ki67, EGFR, PTEN, GFAP, and VEGF). These results suggested that the presence of CTCs was independent of patient and tumor characteristics.

### The presence of postoperative CTCs in glioma patients was a poor prognostic factor

In this study, we initially enrolled 106 patients, and we have follow-up results for 79 patients, with the remaining patients lost contact. The median follow-up time from the end of the operation was 1210 days (range, 208–2140 days). A total of 55 patients (69.6%) experienced disease progression, of which 51 patients died during follow-up (Additional file 1 Figure: S6A). As shown in Additional file 1 Figure: S6B and C, the higher the glioma grade was, the worse the OS and DFS (*p* < 0.0001).

**Fig. 3:**
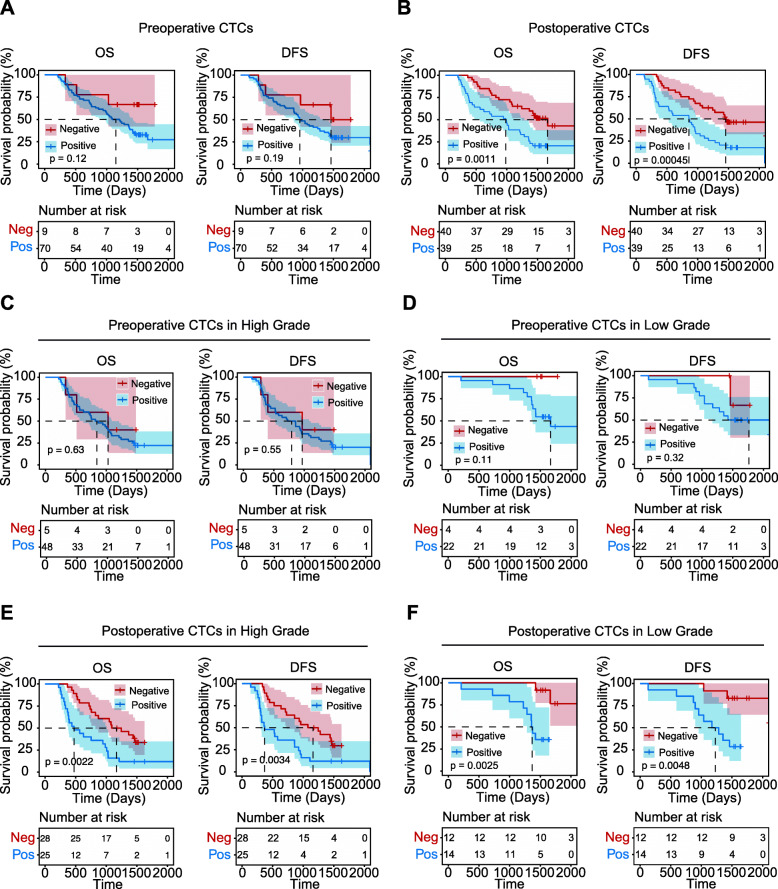
Kaplan–Meier survival curve analysis for enrolled glioma patients. **A** Kaplan-Meier survival curve for OS and DFS of preoperative CTCs. *p*=0.12 and *p*=0.19, respectively. There were no statistical differences between the two groups. **B** OS and DFS of postoperative CTCs. *p*< 0.0001 and *p*=0.0011, respectively. There were significant differences between the two groups. **C** OS and DFS of the high-grade group preoperative CTCs. *p*=0.63 and *p*=0.55, respectively. There were no statistical differences between the two groups. **D** OS and DFS of the low-grade group preoperative CTCs. *p*=0.11 and *p*=0.32, respectively. There were no statistical differences between the two groups. **E.** OS and DFS of the high-grade group postoperative CTCs. *p*=0.0022 and *p*=0.0034, respectively. There were significant differences between the two groups. **F** OS and DFS of the low-grade group postoperative CTCs. *p*=0.0025 and *p*=0.0048, respectively. There were significant differences between the two groups. Red, CTC-negative group; Blue, CTC-positive group. *p* < 0.05 was considered significant, and the 95% CI is shown as a red or blue shadow

Furthermore, according to the results of CTC detection, the patients were divided into a CTC-positive group (CTCs > 1) and a CTC-negative group (CTCs ≤ 1), and survival analysis was conducted for patients in different CTC groups before and after surgery. The median preoperative DFS times were as follows: 956 days (95% CI, 775.6–1136.4) for the CTC-positive group and 1460 days for the CTC-negative group (p=0.19), and the median OS time of preoperative patients was 1089 days (95% CI=804.1–1373.9) for the CTC-positive group (*p*=0.12) (Fig. 3A). However, postoperative CTCs were associated with significantly worse DFS and OS in patients (Fig. 3B). The median DFS was 858 days (95% CI, 466.4–1249.6) for patients with positive CTC detection and 1454 days (95% CI, 965.9–1942.2) for patients with negative CTC detection (*p* = 0.00045), and the median OS was 983 days (95% CI, 636.7–1329.3) for patients with positive CTC detection and 1663 days (95% CI, 1320.4–2005.6) for patients with negative CTC detection (*p* = 0.0011). Furthermore, for both OS and DFS in high-grade and low-grade glioma patients, there were no significant differences between the preoperative CTC-positive and CTC-negative groups (Fig. 3C, D). As shown in Fig. 3E and F, postoperative CTCs were significantly associated with DFS and OS in both low-grade patients and high-grade patients. In the low-grade glioma group, the positive postoperative CTC status was significant (DFS, 1173 days, 95% CI=725.6–1620.4, *p* = 0.0048; OS, 1358 days, 95% CI=1317.7–1398.3, *p* = 0.0025). In the high-grade glioma group, positive and negative postoperative CTC statuses were similar in terms of DFS (368 vs. 1076 days, 95% CI=35.1–700.9 vs. 647.8–1504.2, *p* = 0.00045) and OS (473 vs. 1089 days, 95% CI=192.3–753.7 vs. 694.5–1483.5, *p*=0.0022). The results revealed that postoperative CTCs may be an independent, significant predictor of DFS and OS.

### CTCs that predicted poor prognosis were associated with postoperative neutrophils

**Fig. 4:**
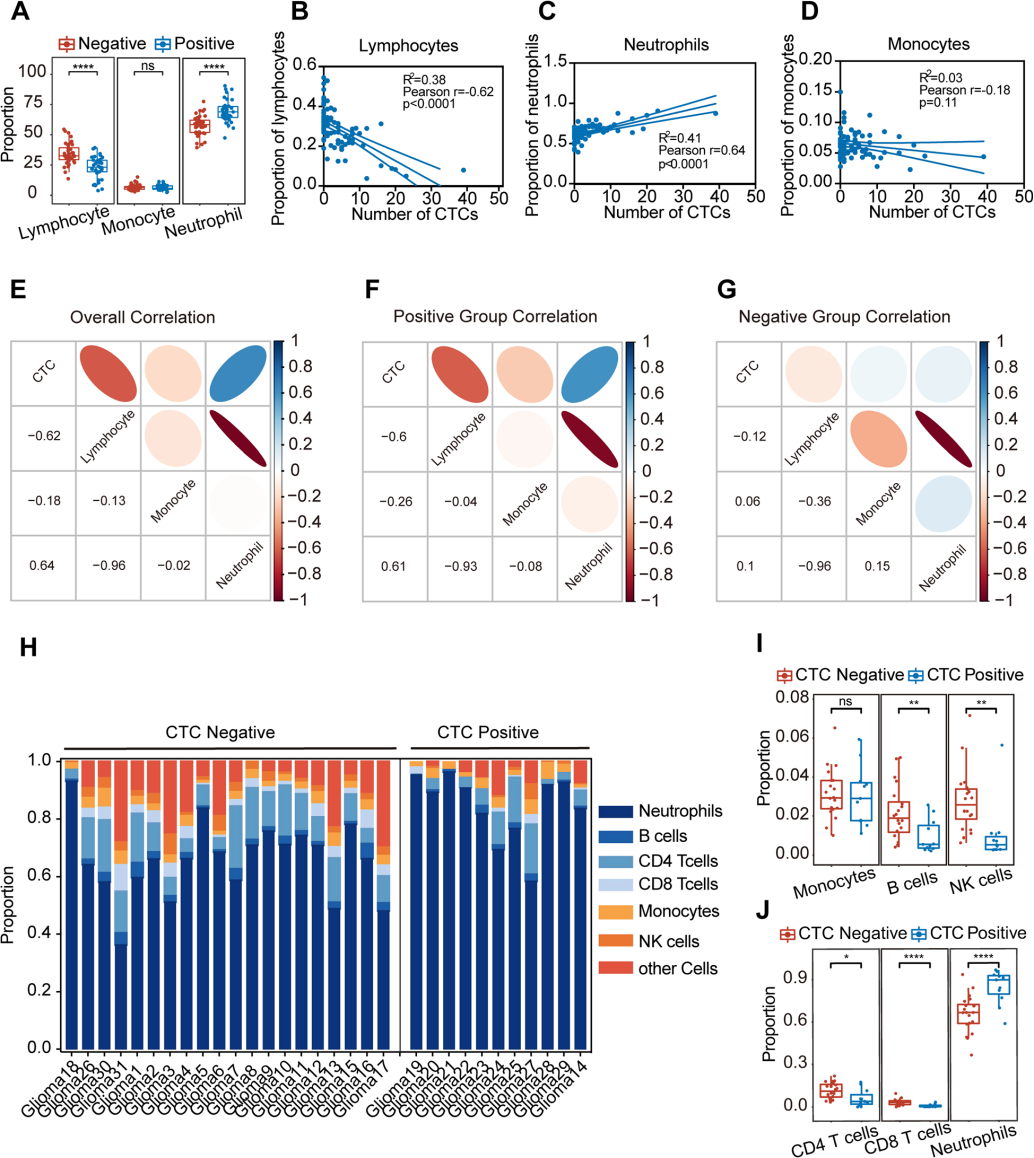
Correlation between postoperative CTCs and clinical blood examination data. **A.** The proportions of lymphocytes, monocytes, and neutrophils between the CTC-negative group and the CTC-positive group. ns, no significant difference, ****, *p*< 0.0001. **B** The correlation between CTCs and the proportion of lymphocytes. Pearson *r*=− 0.62, *p*< 0.0001. **C** The correlation between CTCs and the proportion of neutrophils. Pearson *r* = 0.64, *p* < 0.0001. **D** The correlation between CTCs and the proportion of monocytes. Pearson *r* = − 0.18, *p*=0.11. **E–G**. Correlation heatmap of CTCs and clinical blood examination data in all patients, CTC-positive patients, and CTC-negative patients. Positive correlations are shown in cool colors, while negative correlations (anticorrelations) are shown in hot colors. **H** Cell components of peripheral blood immune cells in the CTC-positive group and CTC-negative group evaluated by EPIC. **I** The proportions of monocytes, B cells, and NK cells between the CTC-negative group and the CTC-positive group. ns, no significant difference, **, *p*< 0.01. **J** The proportions of CD4 T cells, CD8 T cells, and neutrophils between the CTC-negative group and the CTC-positive group. *, *p*< 0.05; ****, *p*< 0.001

To explore the relationship between CTCs and the peripheral immune environment, we further analyzed the correlation between the CTC number and the clinical blood examination data of 79 glioma patients with prognostic information (Additional file 4). Figure 4A shows significant differences in the proportions of lymphocytes and neutrophils between the CTC-negative group and the CTC-positive group (*p*< 0.0001), while there was no significant difference in the proportions of monocytes (*p*=0.45). The correlation between CTCs and peripheral immune cells was further analyzed. Overall, in the 79 glioma patients after surgery, CTCs were significantly negatively correlated with the proportion of lymphocytes (*r* = − 0.62, *p* < 0.0001) and significantly positively correlated with the proportion of neutrophils (*r* = 0.64, *p* < 0.0001), but no correlation was found with the proportion of monocytes (*r* = − 0.18, *p* = 0.11) (Fig. 4B–D). Heatmaps were constructed to show the correlation between the number of postoperative CTCs in the postoperative positive CTC group, the postoperative negative CTC group, and the 79 glioma patients with the clinical blood examination data (Fig. 4E–G). The results of the postoperative CTC-positive group were consistent with the overall correlations, and the postoperative CTC count was positively correlated with neutrophils in CTC-positive patients (*r* = 0.61, *p* < 0.0001). The postoperative CTC-negative group showed no correlations between CTCs and the lymphocyte proportion, neutrophil proportion, or monocyte proportion (Additional file 1: Figure S7). Therefore, the level of postoperative CTCs, which predicts a poor prognosis after surgery, may be associated with neutrophils.

### CTCs are closely related to the activated neutrophil-associated microenvironment of immunity in glioma patients

To verify the correlation between CTCs and the peripheral immune environment, especially neutrophils, RNA sequencing, and TBCD were performed simultaneously using the peripheral blood of 31 glioma patients after surgery. According to the test threshold specified above, the abovementioned 31 patients were divided into a CTC-positive group (*n*=9) and a CTC-negative group (*n*=22) according to the number of CTCs. We first used the EPIC algorithm to predict the cell components of peripheral blood immune cells in different patients, with 15,258 gene results obtained by RNA sequencing (Fig. 4H). As shown in Fig. 4I and G, the proportion of neutrophils in the CTC-positive group was significantly higher than that in the CTC-negative group (*p* < 0.0001), while the other immune cells in the CTC-negative group either were significantly higher than those in the CTC-positive group or were not different (monocytes). Samples with higher CTC counts mainly showed high expression levels of neutrophils, while samples with low CTC counts mainly showed high expression levels of B cells, CD4 T cells, CD8 T cells, monocytes, and NK cells. The results showed that the correlation between the proportion of peripheral blood immune cells evaluated by sequencing results and CTCs was consistent with the correlation between the results of postoperative routine blood examination, and there was a significant positive correlation between CTCs and neutrophils in postoperative peripheral blood.

**Fig. 5:**
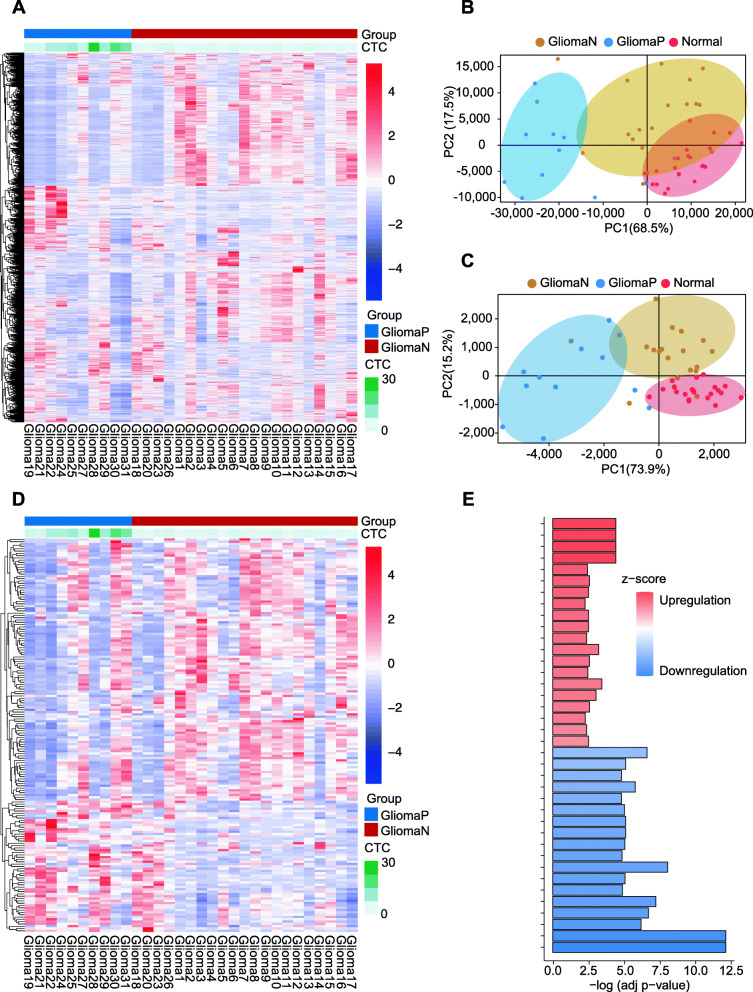
Relationship between postoperative CTC count and macroimmunity in peripheral blood. **A** Gene heatmap of 1729 significantly differentially expressed genes (*p*< 0.05, |log2-fold change|> 1.5) in 31 glioma patients. GliomaP: CTC-positive group, GliomaN: CTC-negative group. **B** Principal component analysis (PCA) of total genes for the CTC-positive group, CTC-negative group, and normal group. Red contour area: normal group, blue contour area: CTC-positive group, yellow contour area: CTC-negative group. **C** PCA of 455 immune-correlated genes with the CTC count for the CTC-positive group, CTC-negative group, and normal group. Red contour area: normal group, blue contour area: CTC-positive group, yellow contour area: CTC-negative group. **D** Gene heatmap of 171 immune-correlated genes (|Cor|> 0.4, *p*< 0.05) in 31 glioma patients. GliomaP: CTC-positive group, GliomaN: CTC-negative group. **E** The most significant Gene Ontology (GO) terms enriched in the genes of interest related to CTC counts. The color of the bar indicates the *z*-score. Upregulation (red, positive *z*-score), downregulation (blue, negative z score).

We further explored the relationship between the leukocyte transcriptome and CTC counts. Using 1729 significantly differentially expressed genes (*p*< 0.05, |log2-fold change|> 1.5) (Fig. 5A), we observed an overall difference in the peripheral blood of the positive and negative groups of glioma patients. PCA was performed in the two groups of glioma patients and the normal group (Fig. 5B). There were differences in the overall differential genes in peripheral blood among the three groups. Next, we identified 455 immunity-correlated genes [18] from 5416 CTC count-related genes (|Cor|> 0.4, *p*< 0.05). The PCA plots showed that the glioma high CTC group was significantly different from the low CTC group in the gene expression profile that was correlated with CTCs, while the negative CTC group was similar to the normal control group (Fig. 5C). The heatmap (Fig. 5D) shows significant differences between the 171 relevant genes in the positive and negative CTC groups (|Cor|> 0.4, *p*< 0.05). The results suggested that the postoperative CTC level was closely related to the postoperative immune status of patients.

When total RNA in the peripheral blood of glioma patients was analyzed by DESeq2, a volcano plot of the immune-related differential genes between the CTC-positive group and the CTC-negative group was obtained (Additional file 1: Figure S8A). Pathway and process enrichment analysis of the differentially expressed genes showed that the genes highly expressed in the CTC-positive group (*p*< 0.05, log2-fold change> 1, *n*=200) correlated with the positive regulation of biological processes such as neutrophil degranulation, neutrophil activation, and neutrophil-mediated immunity (Fig. 5E, Additional file 5). In the CTC-negative group, the highly expressed genes (*n*=252) were mainly associated with T cell activation, lymphocyte differentiation and proliferation, and T cell-mediated immune response (Fig. 5E, Additional file 6). The results indicated that CTCs were positively correlated with peripheral innate immune responses, especially the activation of neutrophils and the generation of neutrophil extracellular traps (NETs), but negatively correlated with the cytotoxic response, especially the T cell cytotoxic response. We speculated that CTC levels in glioma patients might be related to macroimmunity in the peripheral blood of patients.

**Fig. 6:**
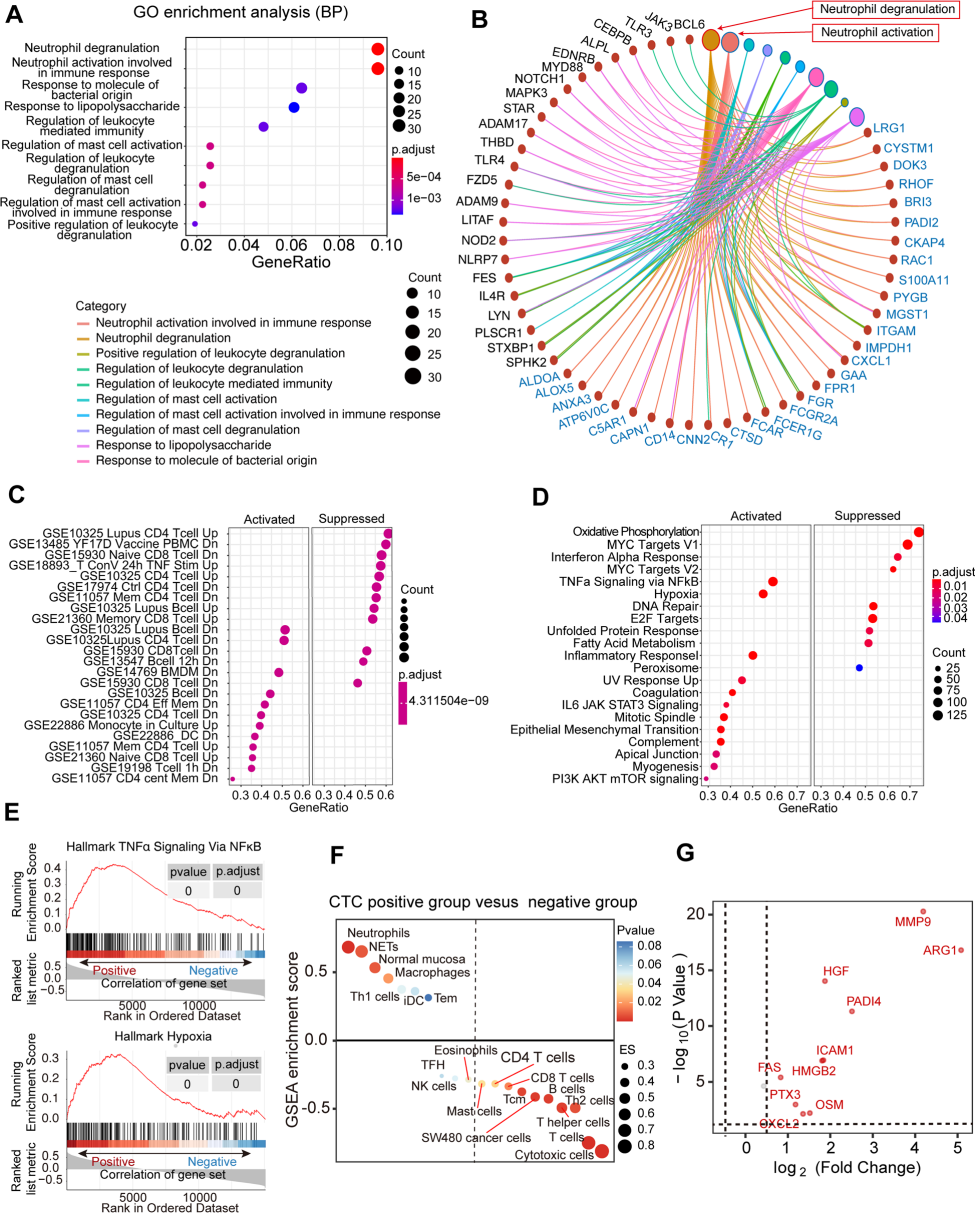
Gene profiles positively correlated with CTC count. **A** The 10 most significant Gene Ontology (GO) terms enriched in the genes of interest. GeneRatio is the percentage of total differential genes in the given GO term. Count is the number of total differential genes in the given GO term. **B** The maximal subnetwork constructed with the genes of interest. The pink nodes represent neutrophil activation, and the second-largest brown node is neutrophil degranulation. The size and color of the edges indicate the coexpression values. Count is the number of total differential genes in the given GO term. **C.** GSEA of glioma sample data (*n*=31) for the c7 immunological gene sets downloaded from the Molecular Signatures Database (MSigDB). GeneRatio is the percentage of total differential genes in the given GO term. Count is the number of total differential genes in the given GO term. **D.** GSEA of glioma sample data (*n*=31) using the hallmark v7.0 gene sets (MSigDB). GeneRatio is the percentage of total differential genes in the given GO term. Count is the number of total differential genes in the given GO term. **E** GSEA of the peripheral blood leukocyte RNA-seq dataset. Activated gene sets showed TNF-α signaling via NF-κB (*p* value< 0.05) with a minimum enrichment score of 0.3 (upper image). Activated gene sets showed TNF-α signaling via hypoxia (*p* value< 0.05) with a minimum enrichment score of 0.3 (bottom image). **F** The relationship between NET scores and gene sets in the CTC-positive and CTC-negative groups. **G** A volcano plot of NET-related differential genes was obtained between the CTC-positive group and the CTC-negative group

To annotate the functions enriched in the 337 genes of interest that positively correlated with CTC levels (Cor> 0.4), Gene Ontology (GO) analysis was performed. The results showed that CTCs positively correlated with the 10 most significantly enriched biological process GO terms and were also associated with neutrophil-mediated immune response, particularly neutrophil activation and degranulation (Fig. 6A, B). In the gene set enrichment analysis (GSEA), CTC count was regarded as a continuous variable. WBCs in patients with higher CTC counts showed a similar trend to the downregulated response of CD4 T cells/B cells instead of myeloid cells (Fig. 6C). The upregulation response of CD4 T cells instead of myeloid cells presented a negative correlation with CTCs (Additional file 1 Figure: S8B and C). Moreover, with a hallmark gene set (Fig. 6D), WBCs in patients with higher CTC counts were activated by TNF-α signaling via NF-κB and hypoxia (Fig. 6E). The activation of these mechanisms can promote the activation of neutrophils, particularly the neutrophil degranulation process, and these changes are consistent with CTC levels. In addition, neutrophilic proteins were analyzed to determine the relationship between NETs formation and tumor progression. GSEA was used to compare the relationship between NETs-related proteins and high and low glioma levels in the CTCs group and showed a difference in tumor transcriptome data between immune-related genes and NETs-related genes (Fig. 6F, Additional file 7). A volcano map of RNA expression based on NETS-related genes (Fig. 6G) is shown in the specific high-low subsets, indicating that patients with elevated CTC levels continued to stimulate increased levels of NETs expression. It can be speculated that the reconstruction of the macroimmune environment by tumor reduction in glioma patients is closely related to CTCs and that the reduction in the macroimmune inflammatory environment can achieve a better prognosis.

## Discussion

In recent years, with in-depth research on the mechanism of tumor metastasis, great progress has been made in the use of CTC detection as a new noninvasive diagnostic method. Although the systemic metastasis of glioma is very rare, some studies have reported the successful isolation and identification of glioma CTCs from peripheral blood [20–28]. The presence of CTCs in gliomas is a common phenomenon, although the reported detection rates vary to a certain extent (20–80%). However, the most prominent difference between central nervous system tumors and epidermal malignant tumors is the lack of EpCAM expression, which makes the detection of glioma CTCs challenging and requires other strategies [27].

In this study, an hTERT-based detection method for circulating tumor cells in glioma patients was established. It has been reported that hTERT expression is increased in commonly used glioma cell lines (U251, U373, and U87) as well as in clinicopathological sections [25, 29]. Based on previous studies, anti-CD45 antibodies can be used to reduce the background of CTC detection and to effectively reduce false positives in CTC detection [30]. Here, for 52 healthy individuals and 106 patients with glioma, the TBCD method was used for glioma sample (ROC) curve analysis. The participants who provided each of the 4-ml blood samples had > 1 under the threshold of CTC, and the sensitivity of the difference between glioma patients and healthy individuals reached 0.830 and 0.865, respectively, showing that the detection method showed very high efficiency.

Compared with other CTC detection methods, the CTC detection method for glioma based on the hTERT promoter has several advantages. First, it does not depend on the expression of tumor cell surface markers, such as EpCAM and GFAP; thus, the CTC tracer is independent of the expression levels of cell surface markers and specific molecular types. Second, since the vector requires infection and replication in active cancer cells to obtain GFP expression, this method labels only viable cells, thus obtaining more reliable CTC assessment results. Although studies have reported high hTERT expression in glioma using an adenovirus vector tracer [25], CTCs can be isolated via our strategy by flow cytometry and enable a further single-cell molecular analysis of informatics analyses to determine more accurate individualized treatment measures for patients.

At present, the reported studies on CTCs of glioma focus on GBM, while there are few studies on other pathological types [20, 27]. In this study, the total detection rate of CTCs in glioma patients was 83.02% (*n*=88/106), which was the highest reported to date, and CTCs of different pathological subtypes were widely present in peripheral blood regardless of the degree of malignancy. This finding suggests that the presence of CTCs is a common feature of gliomas and is not unique to GBM. In the traditional monitoring of clinical response and glioma indicators, pseudoprogression or radiation necrosis may affect treatment judgment and lead to overtreatment [31, 32]. Because CTCs are widely present in peripheral blood, the number of CTCs can indirectly reflect the number and tumor load of intracranial tumor cells. Our results showed that at the 52-month follow-up after surgical treatment, there was a significant positive correlation between postoperative CTC levels and glioma DFS and OS. These results suggest that the CTCs detected by the TBCD assay are independent of the tumor characteristics of the patients and can be used as a supplement independent of other clinical and prognostic indicators.

Subsequently, we analyzed the relationship between systemic immune status and CTCs. In this study, we focused for the first time on CTCs associated with the peripheral immune system as a factor for poor prognosis in glioma. Until now, little has been known about the mechanism by which CTCs rely on the circulatory system to escape the killing attacks of various immune cells, migrate or return to their original sites, and implant there. A recent study has shown that neutrophils are the main white blood cells that interact with CTCs in mouse models and patients [33]. An increasing number of studies have shown that neutrophils play a role in all stages of tumor progression [34, 35]. Allen et al. revealed the remodeling effect of the tumor on systemic immunity through studies on a variety of animal models [36]. Among them, SB28 glioblastoma, located in the brain, has a greater impact on systemic immunity than other tumors. Therefore, through the analysis of the systemic immunity of glioma patients and the correlation between systemic immunity and CTCs, the ability of clinical evaluation and prediction can be further improved.

Although there has been some understanding of the relationship between CTCs and systemic immunity, there are few reports on the association of neutrophils or systemic immune status with glioma CTCs. Our study data showed that postoperative CTC status highly correlated with the overall immunity of the body. On the one hand, the peripheral immune state is characterized by the promotion of the secretion of the inflammatory cytokine TNF-α through neutrophil degranulation or activity enhancement, and TNF-α is associated with the activation of neutrophil extracellular traps (NETs) in the inflammatory response [37, 38]. Regarding glioma patients, the level of CTCs and the expression of NETs-related genes showed obvious convergence, and we thus infer that there is a close relationship between glioma CTCs and NETs, which is associated with a reduction in cell-killing ability, leading to immune escape and a poor prognosis. On the other hand, the analysis of RNA sequencing results also showed T-cell-mediated immune cell damage and decreased lymphocyte immune regulation ability. Additionally, this study showed a correlation between hypoxia and CTCs in the systemic immune state. CTCs in the systemic immune state also face adaptive changes in the hypoxic environment. In the peripheral blood of glioma patients, CTCs significantly correlated with the increased expression of HIF-1α in the systemic immune system. HIF-1α may act as a protective factor for peripheral CTC survival, preventing apoptosis in the peripheral circulation of CTCs and thereby shortening the predicted OS times of patients [39].

Another interesting phenomenon in this study is that although CTCs can be detected in glioma patients both before and after surgery, the patient prognosis was not related to preoperative CTC levels but was significantly related to postoperative CTC levels. There are few studies on the correlation between preoperative CTCs and the prognosis of patients with glioma, but Lynch D. et al. also showed no correlation between CTCs and PFS or OS in a small cohort of patients with glioma [40]. We speculate that CTCs are likely to interact with the immune system, especially the inflammatory response, resulting in a poor patient prognosis. At the tumor site, the inflammatory response is a negative prognostic factor for gliomas [41, 42], and the release of inflammatory factors tends to accelerate glioma progression [43]. However, preoperative tumor-related factors in glioma patients, including pathological grade, lesion site, tumor location, mass size, and edema degree, did not affect inflammatory factors [44]. Therefore, although CTCs exist in glioma patients before surgery, they have no effect on the prognosis of glioma patients due to the systemic noninflammatory immune environment. These results suggest that the systemic immune status should also be considered in the clinical treatment of glioma patients and that targeted systemic immunomodulatory therapy, especially of neutrophils associated with NETs formation, may benefit patients clinically.

## Conclusions

In conclusion, we propose an application to facilitate clinical detection and treatment monitoring for glioma patients by using the TBCD method. This method can stably and easily detect CTCs of a wide range of pathological types of glioma and has the potential to monitor the disease progression of glioma patients, predict prognosis and evaluate the efficacy of individualized treatment. In addition, our results also showed a correlation between CTCs and peripheral immune system immunity, which provides a new perspective for the treatment of glioma.

## Supplementary Information

**Additional file 1.**

**Additional file 2.**

**Additional file 3.**

**Additional file 4.**

**Additional file 5.**

**Additional file 6.**

**Additional file 7.**

## Data Availability

All data generated or analyzed during this study are included in this published article. The raw RNA sequencing data generated in this study are deposited in the Genome Sequence Archive at the BIG Data Center, Beijing Institute of Genomics (BIG), Chinese Academy of Sciences, under the accession number HRA001221 [https://bigd.big.ac.cn/gsa-human/browse/HRA001221].

## Abbreviations

CTCs: Circulating tumor cells
OS: Overall survival
DFS: Disease-free survival
CI: Confidence interval
TBCD: Telomerase reverse transcriptase-based CTC detection method
NETs: Neutrophil extracellular traps
