# Supplementary material for: Telomerase-positive circulating tumor cells are associated with poor prognosis via a neutrophil-mediated inflammatory immune environment in glioma
Source: BMC Med. 2021 Nov 12;19:277. doi: 10.1186/s12916-021-02138-7 (PMC8588721; doi:10.1186/s12916-021-02138-7)
Supplement: Supplementary file 1 — Additional file 1. [file 12916_2021_2138_MOESM1_ESM.pdf]

Additional file 1: Supplementary Figures and legends

1. **Figure S1.** Schematic flow cytometry analysis.
2. **Figure S2.** Verification of TBCD recovery.
3. **Figure S3.** The average number of CTCs in glioma patients vs healthy donors
4. **Figure S4.** Flow cytometry for removal of CD56 background interference.
5. **Figure S5.** The positive rates of preoperative CTCs in different glioma patients' subgroup.
6. **Figure S6.** Kaplan-Meier survival curve for glioma patients enrolled.
7. **Figure S7.** The correlation of the proportions of lymphocytes, monocytes, and neutrophils between the CTC negative group and the positive group.
8. **Figure S8.** Immune-related differential genes between the CTC positive group and the CTC negative group.

Supplementary Fig S1

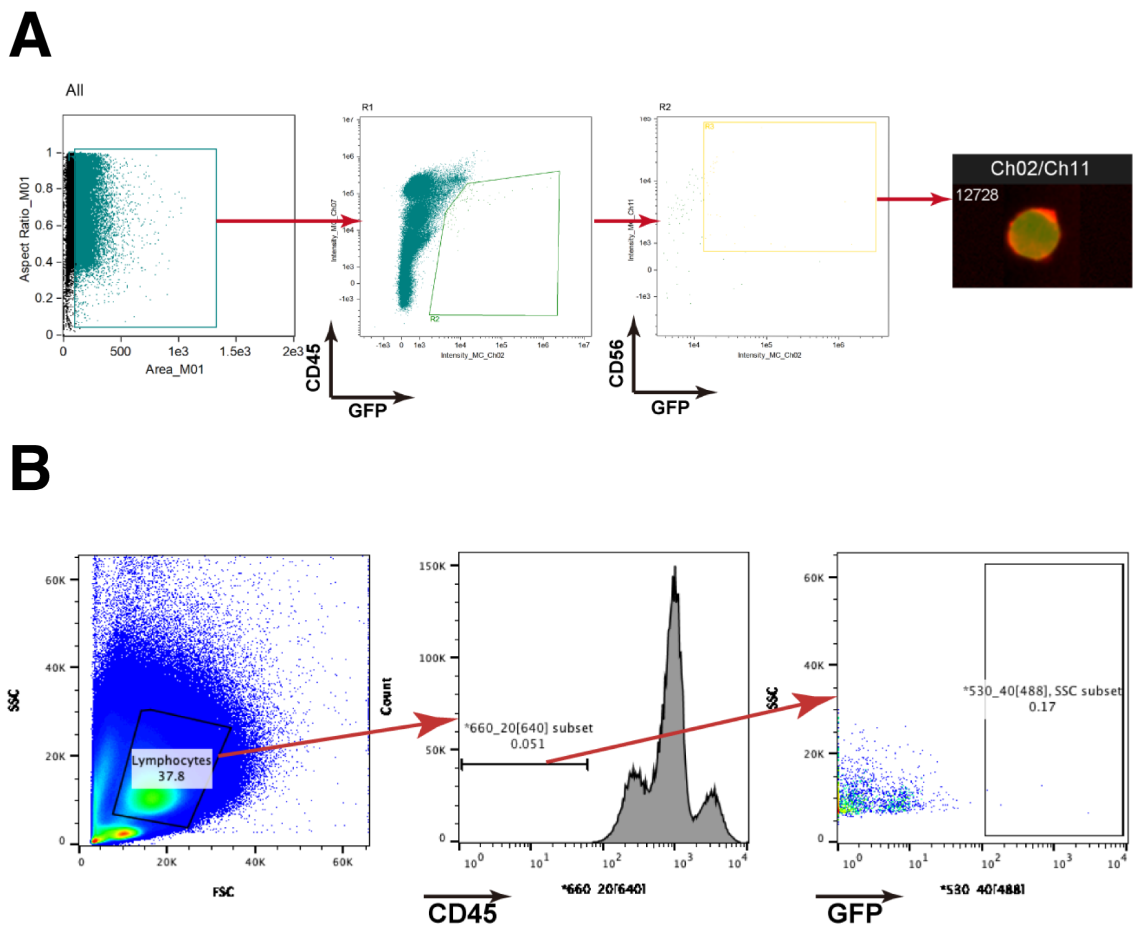

Figure S1. A. Flow cytometry for TBCD analysis. **B.** Flow cytometry for ImageStreamX® system analysis.

Supplementary Fig S2

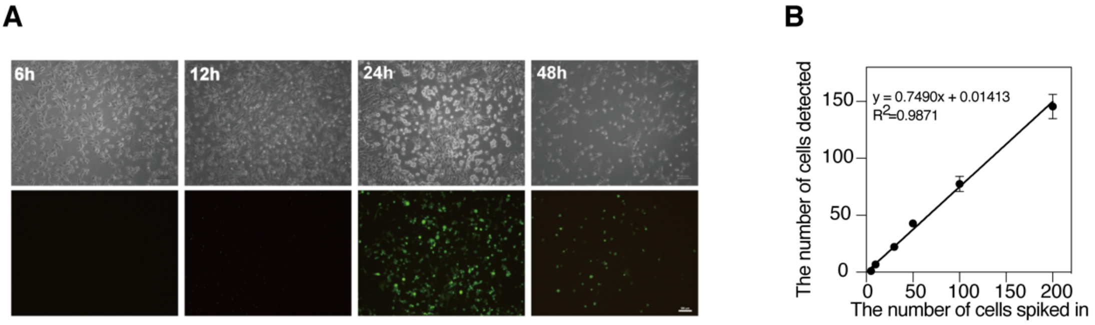

Figure S2. A. oHSV1-hTERTp-GFP selective replication in human glioma cancer cells. Fluorescence micrographs showing GFP expression (bottom panels) of U251 cells 48 hours after oHSV1-hTERTp-GFP transduction at an MOI = 1 and corresponding phasecontrast microscopy images of cell morphology (top panels). Original magnification, ×100. **B.** Accuracy of TBCD in circulating tumor cell (CTC) models of glioma cancer cells. The number of cancer cells spiked into whole blood and the number of GFP-expressing cells detected are plotted. Each value represents the mean ± SD. R2=0.9871.

Supplementary Fig S3

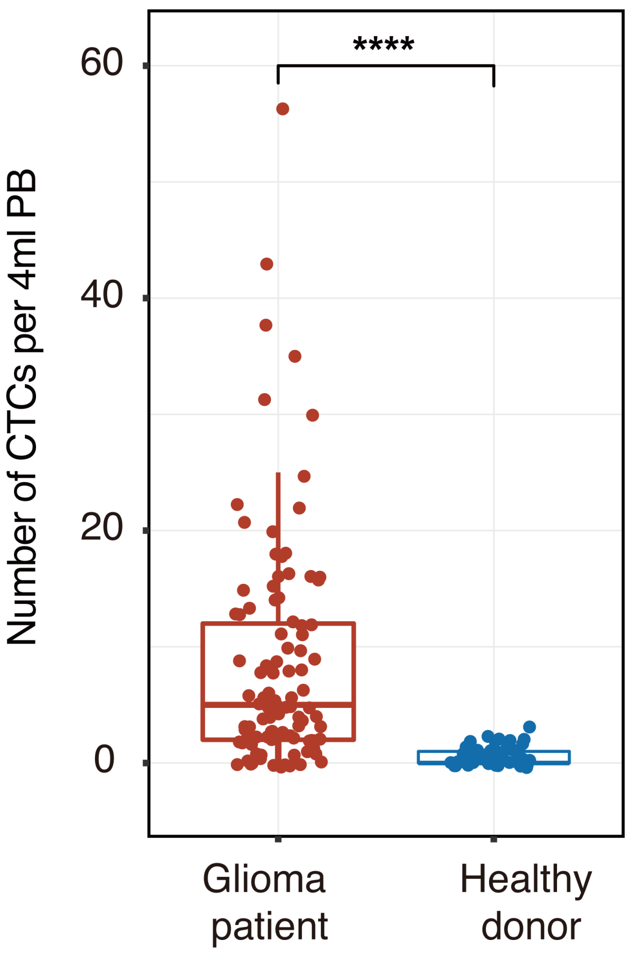

Figure S3. The average number of CTCs in glioma patients vs healthy donors is 8.453 ± 0.955 vs 0.615 ± 0.107 cells/4 ml (****, $p < 0.0001$).

Supplementary Fig S4

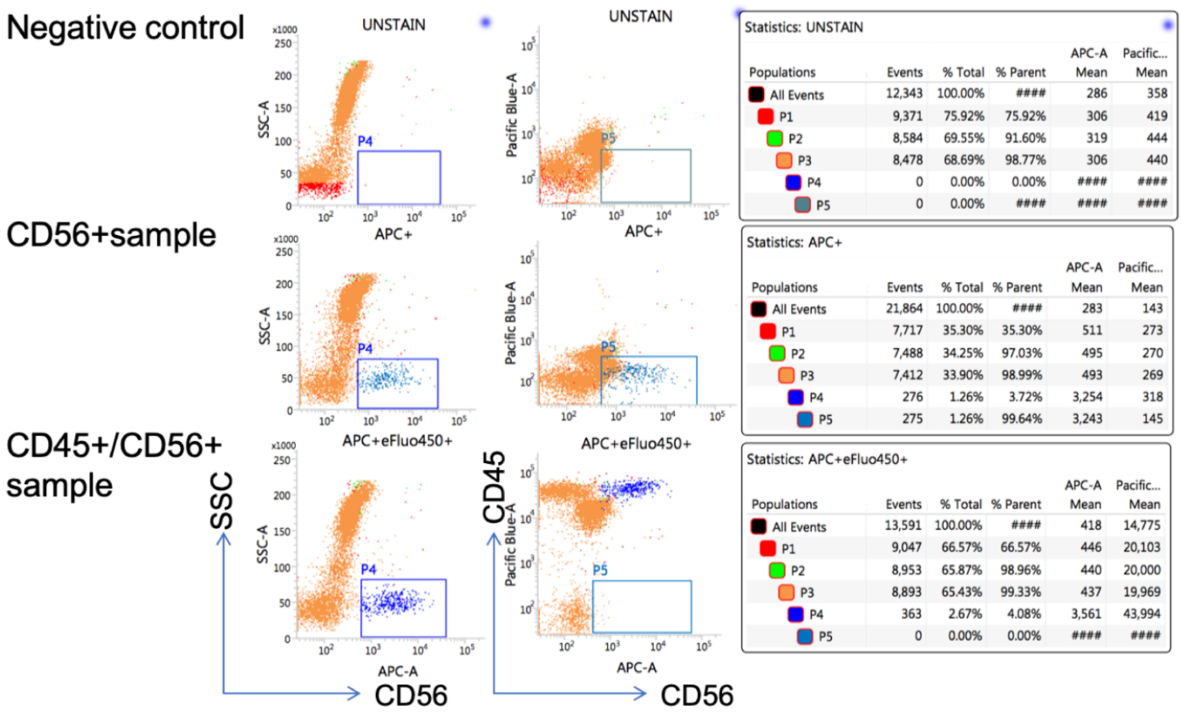

Statistics: UNSTAIN

Populations	Events	% Total	% Parent	APC-A Mean	Pacific... Mean
All Events	12,343	100.00%	####	286	358
P1	9,371	75.92%	75.92%	306	419
P2	8,584	69.55%	91.60%	319	444
P3	8,478	68.69%	98.77%	306	440
P4	0	0.00%	0.00%	####	####
P5	0	0.00%	####	####	####

Statistics: APC+

Populations	Events	% Total	% Parent	APC-A Mean	Pacific... Mean
All Events	21,864	100.00%	####	283	143
P1	7,717	35.30%	35.30%	511	273
P2	7,488	34.25%	97.03%	495	270
P3	7,412	33.90%	98.99%	493	269
P4	276	1.26%	3.72%	3,254	318
P5	275	1.26%	99.64%	3,243	145

Statistics: APC+eFluo450+

Populations	Events	% Total	% Parent	APC-A Mean	Pacific... Mean
All Events	13,591	100.00%	####	418	14,775
P1	9,047	66.57%	66.57%	446	20,103
P2	8,953	65.87%	98.96%	440	20,000
P3	8,893	65.43%	99.33%	437	19,969
P4	363	2.67%	4.08%	3,561	43,994
P5	0	0.00%	0.00%	####	####

Figure S4. Flow cytometry for removal of CD56 background interference. Some NK cells present CD56+. However, CD45 antibodies can be used to negate the white blood cells with high expression of CD56. As shown in the figure, CD45 was used to select all CD56 (NCAM) positive cells, 275 labeled white blood cells in the P5 gate of CD56 samples were observed, and the number of cells in the P5 gate was reduced to 0 after CD45 was added.

Supplementary Fig S5

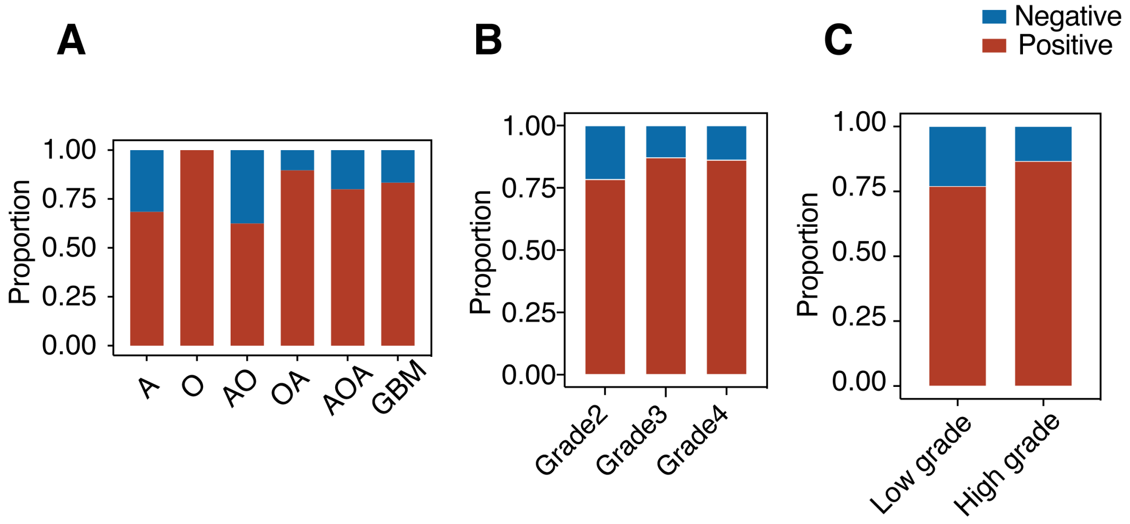

Figure S5. A. The positive rates of preoperative CTCs (range in histogram) for different pathological types. **B.** The positive rates of preoperative CTCs in patients with WHO grade 2–4 gliomas. **C.**The positive rates of CTCs in patients with low-grade and highgrade gliomas.

Supplementary Fig S6

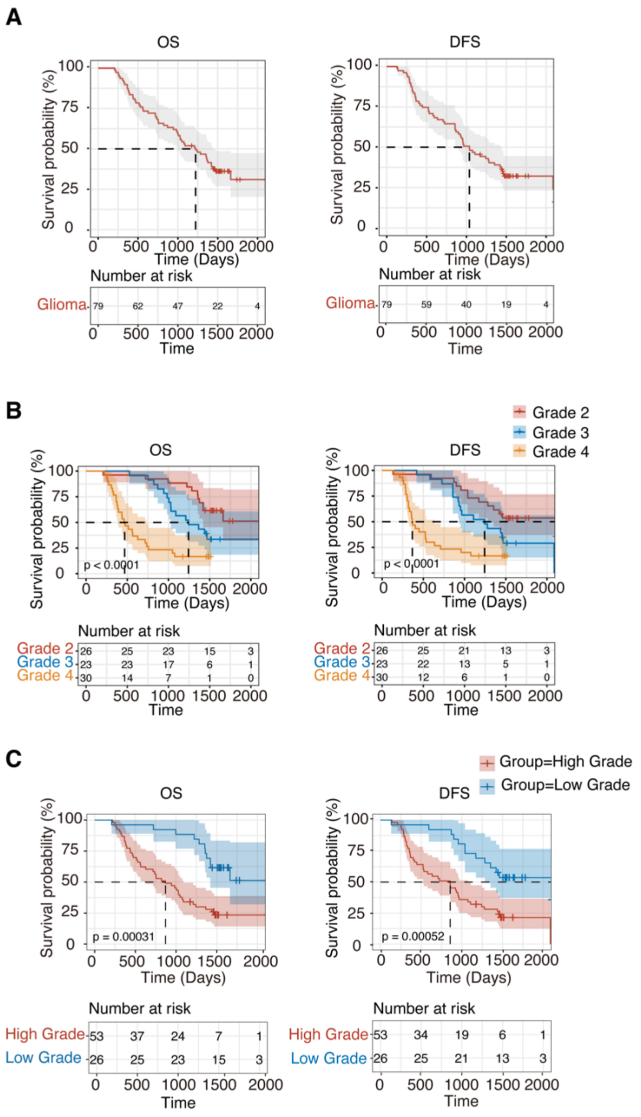

Figure S6. Kaplan-Meier survival curve for glioma patients enrolled. **A.** Kaplan-Meier estimator curve of OS for a total of 79 patients. The median OS was 1223 days. Kaplan-Meier estimator curve of DFS for a total of 79 patients. The median DFS was 1038 days. **B.** Kaplan-Meier survival curve for OS. RED, Grade 2; Blue, Grade 3, Yellow, Grade 4; p<0.0001. Kaplan-Meier survival curve for DFS. RED, Grade 2; Blue, Grade 3, Yellow, Grade 4; p<0.0001. **C.** Kaplan-Meier survival curve for OS of the high-grade group and low-grade group. Red, high-grade group; blue, low-grade group; p<0.0001. Kaplan-Meier survival curve for DFS of the high-grade group and low-grade group. Red, high-grade group; blue, low-grade group; p<0.0001.

Supplementary Fig S7

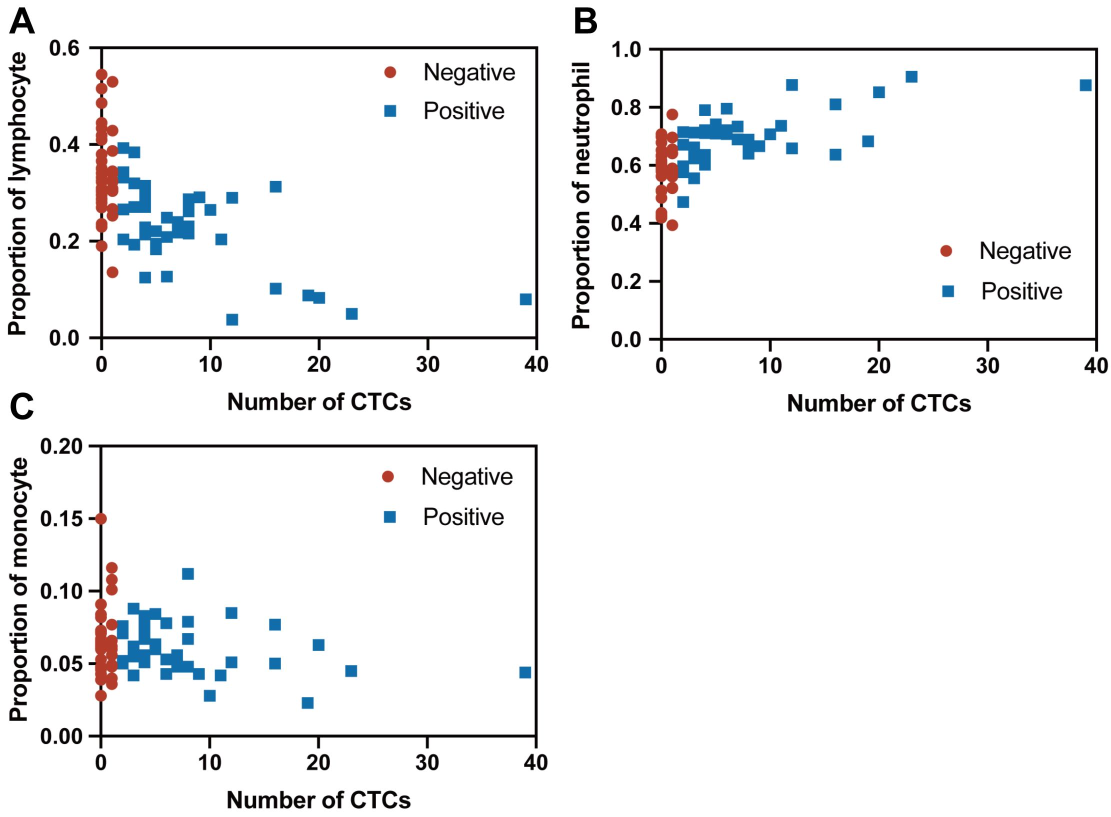

Figure S7. The correlation of the proportions of lymphocytes, monocytes, and neutrophils between the CTC negative group and the positive group. **A.** The correlation between CTC and the proportions of lymphocytes. Red, CTC negative group, Blue, CTC positive group; **B.** The correlation between CTC and the proportions of neutrophils. Red, CTC negative group, Blue, CTC positive group; **C.** The correlation between CTC and the proportions of monocytes. Red, CTC negative group, Blue, CTC positive group.

Supplementary Fig S8

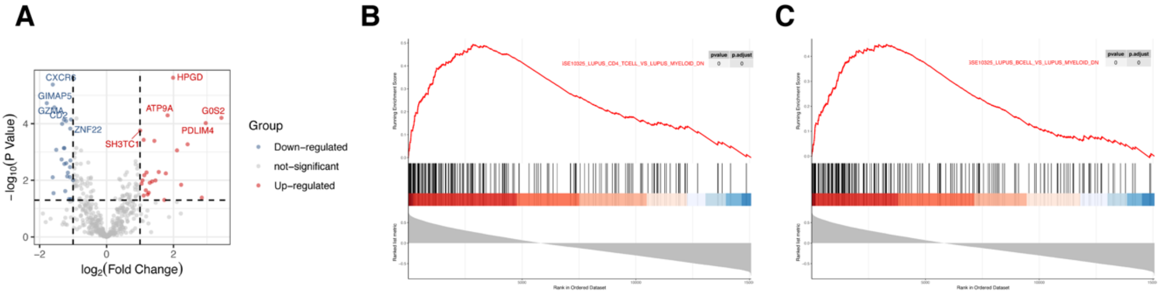

Figure S8. A. A volcano plot of immune-related differential genes was obtained between the CTC positive group and the CTC negative group. **B and C.** GSEA reports show the most 2 activated gene sets for down-regulated lupus CD4 cells vs myeloid and downregulated lupus B cells vs lupus myeloid (pvalue<0.05) with a minimum enrichment score of 0.4.
